# Invasive Candidiasis Coinfection in Patients with Severe COVID-19 Disease: Scoping Review

**DOI:** 10.3390/pathogens14050466

**Published:** 2025-05-10

**Authors:** Omar Esteban Valencia-Ledezma, María del Rocío Reyes-Montes, Gustavo Acosta-Altamirano, María Guadalupe Frías-De-León, Eduardo García-Salazar, Esperanza Duarte-Escalante, Jesús Santiago-Abundio, Zuleyma González-Miguel, María de Lourdes García-Hernández, Rebeca Martínez-Quezada, Oscar Uriel Torres-Páez, Evelyn Galindo-Oseguera, Patricia Meza-Meneses, Nicolás Santiago-González

**Affiliations:** 1 Departamento de Investigación, Hospital Regional de Alta Especialidad de Ixtapaluca, Servicios de Salud del Instituto Mexicano de Seguro Social para el Bienestar (IMSS-BIENESTAR), Ixtapaluca 56530, Mexico; ovalencia.hraei@imssbienestar.gob.mx (O.E.V.-L.); magpefrias@gmail.com (M.G.F.-D.-L.); eduardogs_01@hotmail.com (E.G.-S.); jesus.santiagoab@alumno.buap.mx (J.S.-A.); rebemarq_710@hotmail.com (R.M.-Q.); msptorreshraei@hotmail.com (O.U.T.-P.); egalindo.hraei@imssbienestar.gob.mx (E.G.-O.); dra.patricia.meza.m@gmail.com (P.M.-M.); 2 Departamento de Microbiología y Parasitología, Facultad de Medicina, Universidad Nacional Autónoma de México, Mexico City 04510, Mexico; remoa@unam.mx (M.d.R.R.-M.); dupe@unam.mx (E.D.-E.); 3 Departamento de Investigación del Hospital General de México “Dr. Eduardo Liceaga”, Mexico City 06720, Mexico; mq9903@live.com.mx; 4 Facultad de Enfermería y Obstetricia, Universidad Autónoma del Estado de México (UAEMex), Toluca 50000, Mexico; zgonzalezm@uaemex.mx (Z.G.-M.); mlgarciah@uaemex.mx (M.d.L.G.-H.)

**Keywords:** COVID-19, candidiasis, coinfection, *Candida albicans*, invasive candidiasis

## Abstract

Coinfection rates of candidiasis in patients affected by COVID-19 had a significantly increase during the sanitary contingency. The objective of this scoping review is to analyze the available scientific evidence around the coinfection of invasive candidiasis in hospitalized patients with severe COVID-19 disease. Online databases such as PubMed, EBSCO, SciFinder, Scopus, and SciELO were used to analyze the different scientific studies published from January 2020 to December 2022, selecting 48 publications that reported comorbidity between invasive candidiasis and COVID-19 as a study variable. Based on the PRISMA-ScR extension for scoping reviews, we identified more than half of the publications (57%) as observational, descriptive, and analytic studies, while 43% were systematic reviews. Overall, up to 169,468 adult patients admitted to the intensive care unit were examined. Coinfection was due mainly to *Candida albicans* (75%), but some more species were reported such as *Meyerozyma parapsilosis* (formerly *Candida parapsilosis*); *Meyerozyma guilliermondii* (formerly *Candida guilliermondii*); *Nakaseomyces glabratus* (formerly *Candida glabrata*); *Candida tropicalis*; *Candida dubliniensis*; *Clavispora lusitaniae* (formerly *Candida lusitaniae)*; and *Pichia kudriavzevii* (formerly *Candida krusei*). We concluded that patients infected by SARS-CoV-2 had a higher incidence of fungal coinfections, thus increasing the mortality rate, disease severity, and length of hospital stay in the intensive care unit.

## 1. Introduction

In America, until 2 December 2022, more than 182 million positive cases and almost 3 million deaths were reported according to the Pan-American Health Organization [1]. From the beginning, health providers had faced several challenges, including those related to diagnosis and treatment in secondary infections due to opportunistic pathogens in critically ill patients [2,3]. The Center for Disease Control and Prevention (CDC) pointed out that patients hospitalized due to COVID-19 had a higher risk of healthcare-associated coinfections, in which invasive candidiasis stands out. 

Several studies had described the incidence of candidiasis and mortality rates in patients with COVID-19 as higher in relation to patients without said disease [3,4,5,6,7]. Risk factors related to invasive candidiasis development also associated with poor prognosis were admission to the ICU, the prolonged use of drugs such as antibiotics, steroids, and immunomodulators, and comorbidities like diabetes, lung diseases, or malignancy [3,4]. A study demonstrated that coinfection incidence due to *Candida albicans* was predominantly high in critical patients with COVID-19 in contrast to those without COVID-19 [3], thus founding a higher susceptibility in critical patients [8]. Said fungal infection is considered as opportunistic and had become more frequent worldwide [9]. Fungal coinfections in COVID-19 have a negative impact; in most of the cases of people living in developing countries with low incomes, therefore, a prevention approach must be established [10], necessitating further analysis in fungal coinfection in COVID-19 to improve diagnosis and treatment in order to avoid complications.

Nevertheless, data related to coinfection between COVID-19 and invasive candidiasis are still scarce. Invasive candidiasis continues to be a challenge in healthcare leading to this scoping review in published scientific evidence about the incidence in commonly found species in candidiasis and its association with COVID-19 in order to provide data to help diagnose, treat, and prevent complications in these patients. Based on the aforementioned, the objective in this study was to analyze the available scientific evidence around coinfection with invasive candidiasis in hospitalized patients with severe COVID-19 disease from January 2020 to December 2022.

## 2. Materials and Methods

### 2.1. Study Design 

A scoping review was carried out according to the PRISMA extension for scoping reviews (PRISMA-ScR) [11,12]. The research was carried out in databases such as MEDLINE (PubMed), EBSCO, SciFinder, Scopus, and SciELO. Keywords in DeCS and MeSH included ((((((COVID-19) OR (SARS-CoV-2 Infection)) OR (2019-nCoV Disease)) AND (((Candidiasis) OR (*Candida albicans*)) OR (Invasive candidiasis))) AND ((Prevalence) OR (incidence))) AND (((Coinfection) OR (Comorbidity)) OR (Association))) AND (Adult). Research took into account papers published from January 2020 until December 2022 and limited to Spanish, Portuguese and English. Studies were analyzed if the title and abstract reported comorbidity between candidiasis and COVID-19 as a study variable.

### 2.2. Literature Selection Criteria

Research papers mentioning a coinfection of invasive candidiasis and COVID-19 during hospital stay in adult patients (male and female) were included, if available those with mortality rate comparison. About its study design: clinical trials with more than ten patients, systematic reviews with homogeneity, clinical trials meta-analysis, concurrent cohort studies, systematic reviews of level one diagnostic studies, individual clinical trials with narrow confidence interval and individual concurrent cohort studies with follow-ups higher than 80%. 

Any paper in relation to pediatric population, animal intervention, any other pathogens not related to this study’s approach was excluded, as well as individual cohort studies, low-quality clinical trials, individual case–control studies, case series, and low-quality case–control studies, along with clinical experts’ opinions that lacked explicit critical evaluation. 

### 2.3. Data Collection Process

The research, selection, and review of studies involved was carried out by eight authors (O.E.V.-L., M.R.R.-M., M.G.F.L., E.G.-S., E.D.-E., O.T.-P., P.M.-M., and N.S.-G.) in order to unify coherence between the reviewers. All of them examined the forty-eight papers chosen to be in this scoping review; they analyzed the variables and merged the data extraction before selecting the results. 

### 2.4. Quality Evaluation

To evaluate the quality of these studies, Evidence Classification according to Burns [13] was used. This systematization organizes evidence into a hierarchy of levels from one to five, with one being the “best evidence” and five “the worst or least adequate”. Therefore, in this therapeutics, prevention, etiology, and damage scenario, the best-rated studies correspond to systematic reviews (SR) in clinical Randomized Controlled (RC) Trials. Quality evaluation was carried out individually by each author, later drawing a consensus to sort out any disagreement. 

### 2.5. Data Analysis

Scientific papers were classified based on population, admission area and healthcare center, and design and year lapse; then, epidemiologic data were compiled, as well as the candidiasis characteristics, comorbidities factor, and complications. The inclusion criteria were evaluated in addition to the relation between COVID-19 and invasive candidiasis. The results were showed descriptively, performing a descriptive analysis when the variable allowed for it. 

## 3. Results

### Selection of Sources of Evidence

**Figure 1:**
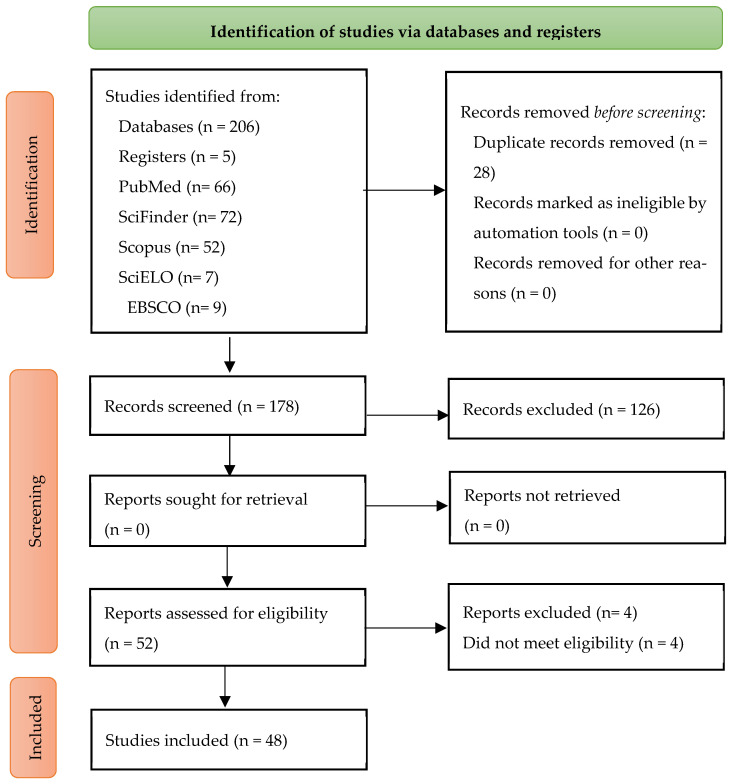
Flux diagram. PRISMA extension for scoping reviews (PRISMA-ScR) [11].

From the scoping review, around 206 papers mentioned the specific subject to analyze in this study. After deleting duplicates, checking the study’s inclusion and exclusion criteria, and performing a conscientious analysis, a sample of n = 48 studies meeting the aforementioned criteria was obtained (Figure 1). 

Table 1 shows the relation of developing candidiasis in patients with COVID-19 where 48 studies were included, of which 43% were literature systematic reviews and meta-analysis, whereas 57% were observational, descriptive, and analytic studies. Said studies included in total 169,468 patients from 17 years old, admitted in the intensive care unit (ICU) in different hospitals around the world. Also, the studies displayed the commonly found *Candida* species, where *C. albicans* was reported in 75% of the cases along with two or more other species.

These studies cover hospitalized patients mainly admitted in the intensive care unit (ICU) in several regions such as Spain, the United Kingdom, Italy, Greece, Hungary, Australia, India, Turkey, Iran, Israel, Africa, the United Arab Emirates, China, South Korea, the United States, and Latin American countries. The studies’ designs include systematic reviews, retrospective studies, cohort analysis, case-series, and transversal studies. Sample size varies from small groups (11 in one paper) to bigger records of thousands of people (47,048 patients in a retrospective study carried out in Spain).

There are several risk factors for developing invasive candidiasis coinfection in patients with severe COVID-19 disease, which are described below: (A) related to COVID-19 infection: prolonged mechanical ventilation, tocilizumab, and steroid (dexamethasone) administration, immunosuppression, and antibiotic therapy, as well as comorbidities such as diabetes mellitus, chronic kidney disease, abdominal surgery, and neutropenia. (B) Related to hospital stay: long stay in the ICU, central venous catheter staying, invasive surgery, and gastrointestinal complications. (C) Patient related: advanced age (>65 years old), and specific COVID-19 immunological alterations, including a high level of pro-inflammatory cytokines and low levels of CD4/CD8 lymphocytes T.

The diagnostic methods used in disease identification—COVID-19 and candidiasis-—are shown in Table 2. The diagnostic method for candidiasis includes the common RT-PCR and blood cultures as standard methods to detect *Candida* spp. In cases of invasive candidiasis, serological tests such as β-D-glucan and mannan antigen are also used. Advance diagnostic methods: MALDI-TOF in rapid species identification, and molecular sequencing techniques (21-plex PCR), specific clinical tests, and histological examination in complex cases. Polymerase chain reaction (PCR) was reported in 40% of all studies, followed by various cultures in 20% and blood culture in 14%.

In the adjacent column, the treatment or therapy during the intervention is described in dosage, duration, and observations related to the therapeutic effect. Immunosuppressors, steroids, antibiotics, azole antifungal drugs, echinocandins, polyenes and antibody therapy are described. In several studies, the association of said therapeutic method and the presence of invasive candidiasis in patients with COVID-19 are mentioned.

The most commonly used drugs include echinocandins (caspofungin, micafungin, and anidulafungin), azole drugs (fluconazole, voriconazole, itraconazole, posaconazole, isavuconazole), polyens (liposomal B-amphotericin), and nystatin, especially in oropharyngeal infections.

## 4. Discussion

Our objective was to describe, according to the extant scientific evidence (2020–2022), coinfection with invasive candidiasis in hospitalized patients with severe COVID-19 disease. Severely compromised COVID-19 patients have a higher probability of developing candidemia due to exposure to risk factors (underlying pathologies and SARS-CoV-2 infection) in the ICU. COVID-19 disease can be complicated by secondary invasive candidiasis.

### 4.1. Coinfection Incidence

Opportunistic mycoses are one of the main factors of severe complications in viral infections due to SARS-CoV-2. Based on the data collected, the incidence rate in patients admitted in the ICU in several hospitals and clinics ranges from 10% to 60% when a COVID-19 diagnosis is present. It is also linked to a high mortality rate (more than 50%) when said coinfection is reported and, in some cases, it reaches 100% mortality with *C. auris* infection, in cases of invasive candidiasis, or when this pathogen is presented in a more aggressive systemic infection [20]. During the COVID-19 pandemic, fungal infection cases increased, especially in those patients with severe viral affection and thus at a higher risk of developing candidemia related to broad-spectrum antibiotics and steroid use; other risk factors include abdominal surgery, mechanical ventilation, parenteral nutrition, or central venous catheter or Foley catheter, on dialysis, with comorbidities (asthma, diabetes, or HIV), or advanced age. Hospital stays in the ICU due to healthcare-related pathogens and the aforementioned procedures are classified as an important risk factor [21,55,56]. The mean age of patients described in the analyzed papers ranged from 50 to 70 years old, thus underlying a more presumptive development of COVID-19 in senior adults in which several factors were not taken into account and representing a higher susceptibility for viral infections [23,29,57,58].

At this moment, when all the data has been compiled, it can be inferred that the pathogens involved in coinfection and increasing the level of lactic dehydrogenase as well as risk factors identified as responsible in the context of COVID-19 and invasive candidiasis coinfection represented a risk in mortality in patients admitted in the ICU. Placing emphasis in infection control through the early detection of both pathogens and treatment, social distancing, maintaining a good hygiene and antifungal prescription improve clinical outcomes when managing this coinfection [15,59,60]. Nowadays, it is well known that SARS-CoV-2 evolution is complicated when secondary infections are presented.

### 4.2. Candidiasis Diagnosis

Presumptive diagnosis in patients admitted in the ICU when COVID-19 is suspected include the rapid antigen test and real-time polymerase chain reaction (RT-PCR) in order to confirmed the viral infection. When talking about invasive candidiasis diagnosis and its several species, the gold standard is the mycological culture also used in most of the analyzed studies. The clinical observation of body areas with candidiasis manifestations reported the tongue as the most common site of infection followed by the soft palate, oropharynx and oral/lips mucosa. Some studies used exfoliative cytology, microbiological cultures, saliva test, and oral mucosa biopsy as diagnostic tools. Candidiasis was confirmed with the presence of yeast and pseudo-hyphae in blood and miscellaneous cultures of each patient. The literature reports symptomatic presentation of candidiasis after the symptoms of COVID-19 are present. Thus, considering sample contamination, to differentiate infection and colonization, the sensibility of the diagnostic tools used and the time when dealing with coinfection were assessed [28,32,38,40,61].

Some studies used serological tests in antigen detection and/or antibodies in blood such as the β-D-glucan test (BDG) and mannan antigen [19,20]; others adopt more sensible techniques and basic tools in clinic microbiology science to confirmed invasive fungal infection.

Yeast isolation in blood culture was reported through the PCR sequencing technique and matrix-assisted laser desorption ionization mass spectrometry with time of fly (MALDI-TOF) [15,41,55].

### 4.3. Candidiasis’ Causal Agent

As part of diagnosis in invasive candidiasis, the commonly found Candida species involved are reported as follows: *C. albicans*, *N. glabratus*, *M. parapsilosis*, *M. guilliermondii*, *C. tropicalis*, *C. dubliniensis*, *C. lusitaniae*, and *P. kudriavzevii*. The most reported species were *C. albicans*, *C. auris*, *M. parapsilosis*, and *N. glabratus*, with *C. albicans* being the predominant causal agent in 75% of all the analyzed studies—also the most frequent organism in invasive candidiasis in patients with COVID-19 [28,37,40,62].

In relation to the persistence pathogens in coinfection, there are several hypotheses remaining unchecked, such as *M*. *parapsilosis* being acquired in external sources, or C. *albicans* and *glabrata* being causal agents that break the normal defense host mechanisms in SARS-CoV-2 infection (epithelial barrier rupture). Other risk factors favor colonization and infection by opportunistic *Candida* spp. commonly found in the human microbiome. For it, the hypothesis of epithelial intestinal interruption in COVID-19 favors yeast migration to deep tissues and organs [55,63,64]. 

### 4.4. Develop and Treatment of Candidiasis

Steroids and immunosuppressor therapeutics increase fungal risk infection, thus increasing mortality rate in coinfected patients. Based on the analysis performed, tocilizumab (TCZ) use could boost the risk to develop systemic candidiasis [14]. TCZ, along with steroid use, was not associated with candidiasis [65]. No association between broad-spectrum antibiotics and Central Line-Associated Bloodstream Infection (CLABSI) due to *Candida* species was found [16]. In case of a fungal indicator in respiratory infections, body fluids are recommended to start an early antifungal therapy based on the patient’s status [18]. Invasive candidiasis treatment is focus in symptoms, signs and culture’s results [21]. A higher mortality rate in late treatment with fluconazole instead of echinocandins as first-line antifungal drugs was presented [55]. International studies reviewed in this article documented that the use of prophylactic antibiotics without strict criteria contributed to intestinal dysbiosis and allowed for the translocation of Candida from mucosal tissues to the bloodstream, causing candidemia. In Iran and India, in addition to candidiasis, there was a surge in mixed fungal infections, such as mucormycosis, partly also related to the overuse of steroids and prophylactic antibiotics. In Spain and Brazil, hospital registries reported that COVID-19 patients who received multiple lines of prophylactic antibiotics had a higher risk of nosocomial fungal infections. In the United States, the CDC warned in 2021 that the overuse of antibiotics during the pandemic could be fueling epidemics of infections by Candida auris, a multidrug-resistant fungus [14,16,21,55,65].

One review in COVID-19 treatments did not mention the antifungal therapy efficacy, since it was uncertain if the mortality was due to the disease itself or fungal infection and its treatment based on early diagnosis [23]. ICU stay, mechanical ventilation, CVC placing, steroid and immunosuppressor therapy were 1.3 times more common in patients with COVID-19, thus increasing hospital mortality [2]. Patients with invasive candidiasis treated with tocilizumab, dexamethasone, continuous renal replacement therapy and ECMO presented more infections and longer ICU stays with ventilation support [6]. B-amphotericin and caspofungin were effective against species of *Candida* and were recommended in pulmonary candidiasis associated with COVID-19 [66]. Steroid drug use is a risk factor that increases mortality in invasive candidiasis associated with COVID-19 [7].

### 4.5. Treatment Duration and Its Effects

All patients affected with *Candida* spp. remained much longer in the ICU in comparison to those who tested negative [14]. Incidence rate in invasive candidiasis associated with COVID-19 was 2.43 per 1000 days in the ICU [2]. Prolonged use of mechanical ventilation and infections were associated with developing invasive candidiasis [67]. Corticosteroids and IL-6 receptor blockers (tocilizumab) were associated with opportunistic infections in COVID-19; thus, its use must be regulated [19]. Fungal infection treatment in critical patients is a challenge due to the comorbidities’ prevalence, toxicity’s risks, and pharmacological interactions [20]. The infection between the host and pathogens must be studied to prompt strategies that helps fight antifungal resistance, scarce in antifungal drugs [68].

The risk of developing invasive candidiasis is higher in advanced-age patients and those with a severe disease [65]. Coinfection with bacteria, drug-resistant pathogens, and several pathogens is associated with higher mortality rates [15]. Patients with COVID-19 and bacterial or viral infections undergoing immunosuppressor therapy had a higher risk of developing opportunistic infections associated with COVID-19: aspergillosis, candidiasis, mucormycosis, cryptococcosis, pneumonia due to *Pneumocystis jirovecci,* and histoplasmosis, amongst others [19]. The increase in invasive candidiasis’s incidence was related to the increase in admissions in patients affected by COVID-19 [17]. Healthcare related fungal infections, in particular due to *Candida* spp., raise the morbidity and mortality rate in critical and severely immunocompromised patients [69]. A high prevalence of candidiasis with COVID-19 disease was identified, about 12% from 889 patients, representing a risk for added infection [24]. In a retrospective study, opportunistic fungal infection was identified to increase mortality rate in patients with COVID-19 [25]. Pulmonary aspergillosis and mucormycosis associated with COVID-19 are the most common fungal infections reported in the literature [4]. Morbidity and mortality rates are linked to invasive candidiasis [5], increasing due to pre-existent conditions, risk factors, and pathophysiological mechanisms [70]. Fungal coinfection between *Aspergillus* spp. and *Candida* spp. are frequent in admitted patients with COVID-19 [71].

### 4.6. Relation Between COVID-19 and Invasive Candidiasis

It is possible that the incidence rate in COVID-19 patients with invasive candidiasis is much higher than reported. All patients positive for *Candida* spp. remained much longer in the ICU compared to those who tested negative; even mortality was also more closely associated with advanced age, coinfection by one or more viruses, bacteria or fungi, influenced by low neutrophil and lymphocyte count and high levels of lactate dehydrogenase. All patients coinfected by COVID-19 and invasive candidiasis were on mechanical ventilation support and CVC placing, receiving broad-spectrum antibiotics, parenteral nutrition, and steroid therapy, reporting an increased incidence of CLABSI due to *Candida* spp., with CVC placing and steroids being risk factors that contribute to fungal infection and a longer stay in the ICU. Meanwhile, factors that worsen COVID-19 evolution/severity include physical barrier and microbiota alterations, vascular catheters, mucositis, GI surgery, immunosuppression, lung disease, steroid or azole use, underlying systemic conditions control and prophylactic and broad-spectrum antibiotics regimen, diabetes mellitus, neutropenia, advanced age, patients with organ transplantation, and invasive candidiasis.

Catheter-related invasive candidiasis was the common entryway in COVID-19 patients, thus increasing morbidity and mortality in severely compromised patients, presenting a high incidence of fungal infection in COVID-19 patients admitted in the ICU. As for early presentation and higher mortality due to invasive candidiasis in said patients, invasive candidiasis coinfection with COVID-19 could worsen the prognosis and recovery.

### 4.7. Study Limitations

This study has several limitations. First, it was a scoping review and did not apply the methodological rigor required for a systematic review, despite using the PRISMA methodology in both cases. This can lead to bias and therefore make a generalization of results impossible. Second, only studies written in Spanish, Portuguese, and English were reviewed. Furthermore, retrospective data published after this study were not considered. Third, we were faced with the lack of methodological models addressing the association between invasive candidiasis and severe COVID-19. This study also has strengths, such as the thorough review and analysis of the literature on invasive candidiasis coinfection in patients with severe COVID-19.

## 5. Conclusions

We concluded that patients infected with SARS-CoV-2 had a higher incidence of invasive candidiasis coinfection, especially due to *C. albicans.* The most common complications were a longer hospital stay in the ICU, higher mortality rate, and a more severe disease in coinfected patients. Management in patients diagnosed with COVID-19 is still deficient and clinical outcomes vary in relation to procedures and treatments. Coinfection according to the clinical pictures is attributed to various reported factors; it is also crucial that in case of evidence of coinfection, the clinical picture is not attributed exclusively to SARS-CoV-2 infection. Diagnostic tests are the biggest challenge, from which it is derived that the care and treatment are the most appropriate and effects of pathogens involved are counteracted. The data collected provide medical evidence to generate approach strategies for invasive candidiasis coinfection in patients diagnosed with COVID-19, as well as to design prophylaxis programs for improving the quality of care for patients admitted to intensive care units due to COVID-19.

## Figures and Tables

**Table 1:** The relation of candidiasis developing in patients with COVID-19 admitted in the intensive care unit in different hospitals around the world, related factors, prevalence, incidence, mortality, and *Candida* species found.

First Author (Year)	Admission	N Patients/Age	Study Design and Settings	Observation Period	Prevalence	Candida Species Found	Risk Factors in COVID-19/*Candida coinfection*
Segrelles-Calvo G. (2021) [14]	ICU	215/>18	Systematic review Observational and prospective study	February–April 2022	Invasive candidiasis 14.4%	*C. albicans* *M. parapsilosis*	All patients were positive to *Candida* spp. Remaining for longer periods in the ICU in comparison to those who tested negative
Jeong S (2022) [15]	ICU	57 admitted 379 outpatient/advanced age	More recent prevalence in coinfection by virus, bacteria and fungi. Observational and prospective study	August 2020– October 2021	Fungal rate 10.5% 6/57	*C. albicans* *M. parapsilosis* *C. tropicalis*	Advanced age Coinfection involving more than one virus, bacteria or fungi Neutrophil and lymphocyte count, as well as lactate dehydrogenase, were associated with higher mortality rate.
Porto Ana P.M (2022) [16]	ITU, ICU	4563/adults	Ecological observational and prospective study	April–June 2020	CLABSI’s incidence: 1.60 (IQR, 0.44–4.20)	*Candida* spp.	Higher incidence of Central Line-Associated Bloodstream Infection (CLABSI) due to *Candida* spp.
Nucci M (2021) [17]	ICU,	41/mean age 62 years	Review study Retrospective	January 2019–February 2020 / March–September 2020	Ranged from 0.7% to 23.5%	*C. albicans*	All patients with candidemia associated with COVID-19 were on mechanical ventilation with a central venous catheter, broad-spectrum antibiotics, steroids, and parental nutrition.
Peman J (2020) [18]	ICU	1095/NR	Review study	2019–2020	NR	*C. albicans* *M. parapsilosis* *C. tropicalis* *N. glabratus* *C. auris* *M. guilliermondii*	Higher levels of pro-inflammatory (IL-1, IL-2, IL-6, TNF-α) and anti-inflammatory (IL-4, IL-10) cytokines. Less IFN-γ, CD4, and CD8 cell expression, thus raising the risk for severe fungi infections.
Abdoli A (2022) [19]	ICU	NR	Review study	NR	NR	*C. albicans* *C. tropicalis M. parapsilosis*, *N. glabratus* *M. orthopsilosis*	Prolonged ICU stay, central venous catheters, and steroid use as main risk factors in fungal infection.
Chiurlo M (2021) [20]	ICU	NR	Review study Systematic review	2020–2021	10% of admitted patients due to COVID-19 infection High mortality rate >50%	*C. albicans* *Candida* spp.	Physical barriers’ alterations, vascular catheters, mucositis, GI surgery, immunosuppression, microbiota alterations, severe lung disease, diabetes or advanced age
Rajendra Santosh A.B. (2021) [21]	ICU	NR	Review study Systematic review	2019–2020	Frequency in infections due to fungi is rising due to the human immunodeficiency virus and immunosuppressive drugs	*Candida* spp.	Organ-transplanted patients, steroid use, azole drug use, control of systemic underlying pathologies, and prophylactic antibiotic regimen. Diabetes mellitus, broad-spectrum antibiotics, neutropenia, steroids, and voriconazole.
Arastehfar A. (2021) [22]	ICU	1988/NR	Ecological observational and retrospective study	November 2020–January 2021	*C. albicans* (57%) *N. glabratus* (28%)	*C. albicans* *N. glabratus* *M. parapsilosis*	Candidemia as a worsening factor in COVID-19 severity, broad-spectrum antibiotics, central venous catheter, mechanical ventilation, IL-6 inhibitor, and tocilizumab use.
Frías-De-León M. G. (2021) [23]	ICU	NR/>40	Bibliographic research	January 2020–February 2021	NR	*C. albicans* *M. parapsilosis* *N. glabratus* *C. tropicalis* *C. auris* *P. kudriavzevii* *C. lusitaniae* *C. inconspicua* *C. dubliniensis* *M. orthopsilosis*	COVID-19 pathophysiology characteristics (high levels of inflammatory cytokines and reduced T-Cells) favor fungal colonization and infection along with mechanical ventilation, central venous catheters, and prolonged hospitalization stay.
Katz J. (2021) [24]	Several hospitals and clinics	889/NR	Using i2b2 database	Year 2019–2021	Disproportionate compromised in Afro-American population (40% of COVID-19 cases and/invasive candidiasis)	*C. albicans*	Invasive candidiasis was associated with a higher risk in COVID-19
Coskun A (2021) [25]	ICU	627/mean of 73.5	Review study Electronic clinical archives	March 2020–February 2021	Ranging from 5% to 70% in mortality due to fungal infection in the ICU	*C. albicans* *M. parapsilosis* *C. tropicalis*	High scores in APACHE II, diabetes mellitus, neutropenia, kidney disease, abdominal surgery, broad-spectrum antibiotics, parenteral nutrition, hemodialysis, mechanical ventilation, central venous catheter, and immunosuppression treatments.
Machado M (2022) [3]	ICU	47,048 on 2019	Retrospective study	January 2019– December 2020	Candidemia’s incidence: 4.73 patients with COVID in 1000 admissions, 0.85 patients without COVID in 1000 admissions	*C. albicans* *M. parapsilosis* *C. tropicalis* *N. glabratus* *P. kudriavzevii* *K. marxianus*	Central venous catheter-related candidemia was the most common entry way for patients with COVID-19
Shishido AA (2022) [4]	ICU	65 /NR	Review study	NR	High mortality rates	*C. albicans*	High incidence and mortality in patients with COVID-19, longer stays in the ICU and CVC longer stay in place, steroids use, sepsis, age higher than 65 years
Szabo BG (2021) [5]	ICU	90 /advanced age (mean of 75.0 ± 13.0 years old)	Case series Retrospective observational study	March–July 2020	*C. albicans 50*%, *N. glabratus* 37.5%, *M. parapsilosis* 12.5% and *M. metapsilosis* 12.5%	*C. albicans* *N. glabratus* *M. parapsilosis* *M. metapsilosis*	Candidemia increases morbidity and mortality in adult patients with severe COVID-19 disease.
Seagle EE (2022) [2]	NR	251/NR	Case analysis	April–August 2020	Up to 25.5% of all patients had a coinfection of *Candida* and SARS-CoV-2	*C. albicans*, *N. glabratus* *M. parapsilosis C. tropicalis* *C. dubliniensis C. lusitaniae* *P. kudriavzevii* *M. guilliermondii*	Patients with COVID-19 had a higher risk of coinfection due to candidemia even when they did not have a commonly associated risk factor for candidemia
Koukaki E (2022) [6]	ICU	178/66	Retrospective observational study	August 2020– November 2021	5 out of 178 patients developed candidemia associated with COVID-19 but only one more patient was affected by candidemia and aspergillosis	*M. parapsilosis* (one patient) *C. auris* (one patient) *N. glabratus*(one patient) *Candida* spp. (three patients)	Higher incidence rate of fungal infections in patients admitted in the ICU due to COVID-19 disease
Erami M (2022) [26]	ICU	69 a 100/61.1 (range = 21–88)	Descriptive study	NR	*C. albicans* (55; 79.7%) *N. glabratus* (12; 17.4%) and two more patients due to (2.9%) *C. africana*	*C. albicans* *N. glabratus* *C. africana*	Infection due to *Candida* spp. did not influence the variables of infection and death due to COVID-19. Airway colonization by *C. albicans* was commonly found, especially in patients with comorbidities such as diabetes, malignancy, and affected by renal alterations.
Kayaaslan B (2021) [7,27]	ICU	2487/72	Retrospective study	March 2020–March 2021	Candidemia’s incidence was higher in the COVID-19 group (2.16, IC 95% 1.77–2.60) than those without COVID-19 (1.06, IC 95% 0.89–0.125)	*C. albicans* *M. parapsilosis* *N. glabratus* *C. tropicalis* and others	Higher incidence and early presentation with increased mortality rate due to candidemia in patients with COVID-19
Salehi M (2020) [27]	ICU	NR	Review study	2020	Until May 25 2020, up to 133,521 confirmed cases of COVID-19 and 7359 deaths were reported in Iran *	*C. albicans*	Inadequate treatment increases the probability to develop a fungal infection, thus increasing the mortality rate.
Vitale RG (2022) [28]	ICU	146/35–88	Review study	2021	Estimated mortality due to invasive candidiasis ranged from 19% to 40% and up to 70% in the ICU	*C. albicans* *C. auris* *N. glabratus* *C. tropicalis* *M. parapsilosis* *C. dubliniensis* *M. orthopsilosis* *P. kudriavzevii*	In patients with COVID-19, fungal infections could worsen the prognosis and recovery
White, PL (2021) [29]	ICU	51/mean age of: 57, M/F: 2.2/1	Evaluation of a prospective cohort study	NR	Incidence of 26.7% (14.1% in aspergillosis and 12.6% in invasive candidiasis)	*C. albicans*	Invasive fungal disease associated with COVID-19
Salehi M (2020) [30]	Several hospitals and clinics	53/27 to 90	Transversal study	March 2020– April 2020	During the study, up to 53 (5%) out of 1059 iranian patients with COVID-19 confirmed infections had OPC*	*C. albicans* *N. glabratus* *C. dubliniensis M. parapsilosis* *M. tropicalis* *P. kudriavzevii*	* Invasive candidiasis (OPC) in patients with COVID-19
Senok, A. (2021) [31]	Dubai’s hospital electronic system	29.802/49.3 ± 12.5	Retrospective review	February 31–July 2020	1.3% presented coinfection	*C. auris* *M. parapsilosis*	Coinfection in patients with COVID-19
Sang, L. (2021) [32]	Several hospitals and clinics	190/NR	Retrospective review of medical records of adult patients	January 2020 –April 2020	*C. albicans* (6.8%)	*C. albicans*	Secondary infection in patients with COVID-19
Jeong, S. (2022) [15]	Several hospitals and clinics	436 samples of 57 admitted patients and 379 outpatients/65.7% were >60 years old	Prevalence evaluation in coinfection due to virus, bacteria and fungi in patients with COVID-19	August 2020–October 2021	Incidence rate in coinfections due to bacteria or fungi were 52.6% and 10.5%, respectively, in patients admitted due to COVID-19	*C. albicans*	Higher coinfection rate in patients with COVID-19 disease
Mastrangelo A. (2021) [33]	ICU	72/NR	Prospective cohort study comparing historical control patients without COVID-19	February 2020–June 2020	35 (48.6%)	*C. albicans*	A characteristics description of candidemia in patients affected by SARS-CoV-2
Amorim dos Santos J (2021) [34]	Worldwide study	64,876/NR	Systematic review	January 2021, six months after the initial research (June 2020)	Eight studies reported invasive candidiasis	*C. albicans*	It reported oral signs and symptoms in patients with COVID-19 disease
Roudbary, M. (2021) [35]	Several hospitals and clinics	NR	Literature research	Between 2020 and 2021	Common fungal infections were invasive candidiasis and aspergillosis	*C. albicans*	It reported opportunistic fungal diseases in patients with COVID-19 disease
Denny S. (2021) [36]	Several hospitals and clinics	11/> 17 years old	Retrospective review in candidemia	March 2020–May 2020	*C. albicans* in 63.6%	*C. albicans* *M. parapsilosis* *N. glabratus* C. *dubliniensis*	It describes the high incidence of candidemia in patients with COVID-19 disease
Norberg, C M. (2021) [37]	Scientific literature analysis, different regions of the world	NR	Bibliographic review	2021	8 out of 9 patients had a coinfection due to *Candida* spp. (*N. glabratus* (4), *C. auris* (3) and *C. albicans* (1)	*C. auris*	Despite the high risk of developing fungal coinfection in patients infected by SARS-CoV-2, the data are scarce in relation to incidence and risks of secondary infections
Samaranayake, L. P. (2022) [38]	Database (Pubmed, OVID, SCOPUS and Web of Science)	292/NR	Systematic review	March 2020– October 2021	*Candida* infection was the most common coinfection, 64% (n = 96)	*C. albicans*	Orofacial mycoses in COVID-19 disease
Brandi, N. (2022) [39]	ICU	95/NR	One center observational and retrospective study	October 2020–January 2021	27 (42.9%) patients tested positive for bacterial and fungal infections and 3 patients (4.8%) were affected exclusively by fungi	*Candida* spp.	Fungal coinfections are frequent in patients with COVID-19 admitted in the ICU and are associated with poor outcomes
Rafat, Z. (2022) [40]	ICU	73/NR	Transversal study in which sputum samples and endotracheal aspirate of patients with COVID-19 in the ICU were collected	May to October 2020	15 cases (20.5%) confirmed with fungal coinfections	*C. albicans*	Patients with severe COVID-19 disease in the ICU were prone to develop fungal infections
Ayalon, O. (2022) [41]	ICU	311/NR	Case–control study	1 September 2020–31 March 2021	Candidemia 3.5%	*C. albicans*	Incidence of invasive candidiasis in patients with COVID-19 disease
Soltani S. (2021) [42]	NR	2246 patients	Systematic review and meta-analysis	1 December 2019–30 December 2020	Grouped prevalence of fungal coinfection 12.6%	*Aspergillus* 2.39% *Candida* 0.39%	NR
Kamali Sarvestani (2021) [43]	ICU	153 patients	Transversal review	March 2020–March 2021	NR	*C. albicans* (7/12, 58.3%), *C. dubliniensis* (2/12, 16.6%), *C. tropicalis* (1/12, 8.3%), *N. glabratus*(1/12, 8.3%), *P. kudriavzevii* (1 /12, 8.3%)	Presence and treatment of candidemia due to C. *albicans* and related species (*C. dubliniensis*) in Iranian patients with COVID-19
Kubin CJ. (2021) [44]	Manhattan, New York, EEUU	516/3028 patients	Retrospective cohort study	2 March and 31 May 2020	NR	NR	Fungal infections manly due to healthcare-related *Candida* spp.
Cataldo MA (2020) [45]	ICU	2	Retrospective cohort study	March–April 2020	The incidence of invasive candidiasis in patients admitted in the ICU was higher in those affected by COVID-19 than prior the pandemic	*C. albicans* *M. parapsilosis N. glabratus*	Patients with COVID-19 had a higher risk to develop candidemia during stay in the ICU
Agrifoglio A (2020) [46]	ICU	139	Retrospective analysis	February to June 2020	The four months candidemia incidence was 10.8%, much higher in comparison to the seven years prior data	*C. albicans* *M. parapsilosis N. glabratus*	It was identified an exponential raise in invasive candidiasis cases
Hughes S (2020) [47]	Several hospitals	836	Observational study	February 20– April 20 2020	The incidence of bacterial and fungal coinfection was observed in patients admitted with severe acute respiratory distress syndrome	*C. albicans*	The main pathogen involved in fungal coinfection was *C. albicans*
Antinori S. (2020) [48]	ICU	99	Review article	2020 and January 2022	NR	*N. glabratus* *C. albicans*	Evidence reveals bacterial and fungal coinfection in COVID-19 patients
Papadimitriou-Olivgeris M. (2022) [49]	ICU	3572	Retrospective study	2010–August 2021	Steroid therapy was evaluated in relation to develop candidemia during the COVID-19 pandemic	*M. parapsilosis* *C. auris*	A significant increase in candidemia incidence was evaluated during the COVID-19 pandemic in patients with and without COVID-19
Baddley JW. (2021) [50]	ICU	37 studies	Retrospective study	June 2021 and November 2021	Fungal coinfection’s incidence varies and it is related to the population heterogeneity, surveillance protocols, and fungal infection definition	Invasive candidiasis and endemic mycoses	Invasive fungal infections are associated with severe lung injury and immunological deficits such as HIV or immunomodulatory drugs
Macauley P. (2022) [51]	ICU	3568	Overall analysis and comparison	May 2021 and October 2021	12 cases in COVID-19 group (5.1% incidence) 51/1.000 admissions	*C. albicans* accounted for a minority of isolates	Increase in cases in the SARS-CoV-2 pandemic.
Basile K. (2022) [52]	ICU	Not specified	Review article	6 December 2021 and 6 January 2022	Not specified	*Aspergillus* fungal infections including invasive candidiasis, cryptococcosis, pneumocystosis, mucormycosis, and endemic mycoses	Increase in fungal infection associated with COVID-19 disease
Kayaaslan B. (2021) [7]	ICU	1229	Retrospective study	August 2020 to August 2021	The candidemia incidence was evaluated in critical patients affected by COVID-19 with risk factors	*C. albicans*	Patients with severe COVID-19 disease had a higher risk of developing candidemia due to exposure to classical risk factors and specific risks in COVID-19 in the ICU
Elbaz M- (2022) [53]	ICU	1000	Multicenter Cohort Study	February 2020 and May 2021.	Variation in incidence of lung disease due to mold, ranging between 0 and 51.2 per 1000 critical hospitalizations.	Lung disease due to mold associated with COVID-19 and invasive candidiasis	Very variable data on mold conditions have been reported.

Source: Scoping review database about the relation between COVID-19 and invasive candidiasis. N= 48 studies. NR: not recorded; ICU: intensive care unit; CLASBI: Central Line-Associated Blood Stream Infections; OFC: Orofacial Candidiasis; * OPC: Oropharyngeal Candidiasis; APACHE II: Acute Physiology and Chronic Health Disease Classification System II; IL: interleukin; INF: interferon gamma.

**Table 2:** Diagnostic-type descriptions of COVID-19, invasive candidiasis coinfection, and pharmacological therapeutic effect.

First Author (Year)	Country	Diagnosis Type COVID-19	Diagnosis Type Invasive Candidiasis	Treatment
Segrelles-Calvo G. (2021) [14]	Spain	PCR and IgG	Blood culture	Immunosuppressant/anti-inflammatory (tocilizumab (TCZ)) immunosuppressants/systemic corticosteroids (tocilizumab and systemic steroids (SS)) immunomodulator/antiviral (interferon 1β (IFN-1 β)) antiviral (lopinavir–ritonavir)
Jeon S (2022) [15]	United Kingdom	NR	RT-PCR multiplex Matrix-assisted laser desorption ionization mass spectrometry (Vitek-MS) (MALDI)	Immunosuppressors including steroids and TCZ
Porto Ana PM (2022) [16]	Brazil	NR	NR	Antibacterial (piperacillin–tazobactam (PIP-TZ) meropenem and vancomycin)
Nucci M (2021) [17]	Brazil	NR	NR	Antifungal (anidulafungin and fluconazole)
Peman J (2020) [18]	USA Brazil, India, Russia, Peru, Chile, Mexico y South Africa	NR	NR	Antifungal (anidulafungin and isavuconazole)
Abdoli A (2022) [19]	NR	NR	Serological test with β-D-glucan (BDG) and mannan antigen	Echinocandins Azoles (voriconazole/fluconazole/ posaconazole/isavuconazole) Polyenes (liposomal amphotericin b)
Chiurlo M (2021) [20]	NR	RT-PCR-antigen test	Pathogen isolation serological test with β-D-glucan (BDG) and mannan antigen	Immunosuppressor drugs, TCZ use, steroids, and anti-IL-6 receptor agents
Rajendra Santosh AB. (2021) [21]	India	NR	Exfoliative cytology Pathogen culture Saliva test and oral mucosa biopsy	Polyenes (nystatin and b-amphotericin) Azoles (fluconazole, itraconazole and pozaconazole) Antimetabolites (flucytosine)
Rajendra Santosh AB. (2021) [54]	India	NR	Special care must be given to patients with a recent diagnosis of COVID-19 to detect and prevent mucormycosis	NR
Arastehfar A. (2021) [55]	Iran	RT-PCR	Positive blood culture, 21-plex PCR and sequencing	Antifungals (fluconazole or caspofungin)
Frías-De-León M. G. (2021) [23]	Several areas	RT-Q PCR	Molecular and microbiological	Azole antifungal drugs (fluconazole, voriconazole, isavuconazole) echinocandins (caspofungin, anidulafungin, micafungin) Polyenes (b-amphotericin, nystatin)
Katz J. (2021) [24]	Africa	NR	NR	NR
Coskun A (2021) [25]	Turkey	COVID-19 through electronic medical records and blood cultures	Blood culture	Carbapenem and glycopeptides 27 remaining patients with combination of carbapenem and oxazolidinone or glycopeptide family drug
Machado M (2022) [3]	Spain	PCR	Blood culture	Antifungal (echinocandins and fluconazole)
Shishido AA (2022) [4]	NR	NR	NR	Steroids and immunosuppressor therapy
Szabo BG (2021) [5]	Hungary	PCR	Blood culture	Antifungals (caspofungin, fluconazole, voriconazole, itraconazole, isavuconazole, B- amphotericin)
Seagle EE (2022) [6]	USA	PCR	Blood culture	NR
Koukaki E (2022) [6]	Greek	PCR	Blood culture	Half of all patients were treated with TCZ and a high dose of dexamethasone, two more received additional monoclonal antibody therapy
Erami M (2022) [26]	Iran	Diagnosed based on symptoms, radiological signs, PCR	Microbiological tests	Steroid dosage > 2 mg/kg dexamethasone Antifungals (b-amphotericin, voriconazole, itraconazole, fluconazole, caspofungin)
Kayaaslan B (2021) [7]	Turkey	PCR or common finding of COVID-19 in CT-SCAN with a positive antigen test	Blood culture	Antifungals (fluconazole, voriconazole, caspofungin and micafungin)
Salehi M (2020) [27]	Iran	Physical examination and PCR	Blood culture, MALDI-TOF (blood culture) and RT-PCR	Broad-spectrum antibiotics, immunosuppressors or steroids, invasive or non-invasive mechanical support Antifungals (fluconazole and nystatin)
Vitale RG (2022) [28]	India, Brazil, China, Italy, Iran, UK, USA, Mexico, Colombia	PCR	Blood culture	Antifungal and antibiotic treatment Steroids Antifungals (B-amphotericin, anidulafungin, liposomal, isavuconazole, micafungin, voriconazole)
White, P. L, 2021 [29]	NR	PCR	PCR for *Pneumocystis* NBL-BAL. Serological BDG proposed if positive more test should be run for fungi (PCR- GM-EIA)	Antifungal therapy (AFT) in this cohort study could be beneficial for survival if started early, but it needs prospective validation. Prophylactic AFT could be beneficial in this group
Salehi M. (2020) [27]	Iran	PCR and sequencing technique of internal transcribed spacing region (ITS1–5.8S-ITS2)	Presence of gemmating yeast and pseudo-hyphae in a 10% KOH preparation and culture	Most of the isolated *Candida* species were sensible for three antifungal drug families: azole (fluconazole, voriconazole and itraconazole), polyenes (B-amphotericin), and echinocandins (caspofungin, anidulafungin and micafungin).
Senok, A. (2021) [31]	Arab Emirates United	RT-PCR test for SARS-CoV-2	Coinfection confirmed by laboratorial test and culture	Mean lapse to empiric antibiotic star was 1.2 ± 3.6 days after admission, with ceftriaxone, azithromycin, and piperacillin–tazobactam being the most common used drugs
Sang, L., (2021) [32]	China	NR	Bacterial and fungal frequency was measured in cultures of airway and blood samples	Antifungal and antibiotic treatment was administrated in 71 (43.8%) patients
Jeong, S., (2022) [15]	South Korea	PCR-RT	Culture with antibiogram to detect pathogens were carried out and underwent a Multiplex test	Poor immune response due to SARS-CoV-2 infection and immunosuppressor treatment (steroids and tocilizumab) and COVID-19 therapeutics may boost fungal infection
Mastrangelo, A. (2021) [33]	Italy	NR	NR	Antifungal
Amorim dos Santos J, (2021) [34]	Worldwide	PCR	Invasive candidiasis infection was confirmed in presence of germ tube; positive presence of pseudo-hyphae in a 10% KOH preparation and culture	NR
Roudbary, M., (2021) [35]	Several regions worldwide	PCR	MALDI-TOF (blood culture) Molecular sequencing technique	Antifungals (intravenous fluconazole, caspofungin, micafungin, anidulafungin, and b-amphotericin)
Denny, S. (2021) [36]	United Kingdom	PCR	Blood culture identified throughout spectroscopy	All isolated pathogens were sensitive to fluconazole, with the exception of one case, *N. glabratus*, that showed moderate sensibilization
Norberg, C. M. B. M. (2021) [37]	Brazil	PCR	Blood culture IgG test and germ tube test	An association exists between tocilizumab treatment and the development of candidemia in patients with COVID-19
Vitale, R. G. (2022) [28]	Brazil	PCR	Culture	In Brazil, all species of *C. auris* were reported as sensible to azole, amphotericin, and echinocandins
Samaranayake, L. P. (2022) [38]	14 countries	PCR	Clinical observation of sites with systemic candidiasis manifestation	Infections due to *Candida* spp. were treated with antifungals (oral nystatin, miconazole, or systemic fluconazole)
Brandi, N. (2022) [39]	Italy	PCR	Radiological images are a key component to detect coinfections	Non-specific therapy recorded
Rafat, Z. (2022) [40]	Iran	PCR	Direct microscopic observation 10% KOH preparation and culture	NR
Ayalon, O. (2022) [41]	Israel	PCR	Spectometry MALDI-TOF (blood culture)	NR
Salehi M (2020) [27]	Iran	PCR	Sequencing technique of internal transcribed spacing region (ITS1-5.8S-ITS2) and microbiological methods	Antifungals (fluconazole, itraconazole, voriconazole, B-amphotericin, caspofungin, micafungin, and anidulafungin)
Soltani S. (2021) [42]	Iran	NR	NR	Antifungals (amphotericin B, micafungin, and fluconazole)
Kamali sarvestani h. (2021) [43]	Iran	PCR	Blood cultures, mycological test and sequencing technique of internal transcribed spacing region	Antifungals (caspofungin alone, B-amphotericin, voriconazole, fluconazole, itraconazole)
Kubin CJ. (2021) [44]	USA	PCR	Blood cultures	Hydroxychloroquine, azithromycin, low-dosage methyl-prednisone, and fluconazole. Remdesivir (antiviral), vancomycin, and carbapenem (antibacterials).
Antinori S. (2020) [48]	Italy	NR	NR	Antifungals (voriconazole, voriconazole switched to isavuconazole, isavuconazole, caspofungin followed by voriconazole, liposomal amphotericin B)
Papadimitriou-olivgeris M. (2022) [49]	NR	NR	NR	Antifungals (fluconazole, voriconazole, echinocandins, anidulafungin, caspofungin, micafungin, liposomal-amphotericin b)
Basile K. (2022) [52]	Australia	PCR	Blood cultures	Antiviral Treatment
Kayaaslan B. (2022) [7]	Turkey	NR	NR	NR
Elbaz M. (2022) [53]	Israel	PCR	Blood cultures	NR

Source: Scoping review database in coinfection between invasive candidiasis and COVID-19 in patients with clinical complications. NR: not recorded; TCZ: tocilizumab; SS: systemic steroids; INF 1B: interferon 1β; LPV-RTV: lopinavir–ritonavir; PIP-TZ: piperacillin–tazobactam; PCR-RT: polymerase chain reaction in real time; MALDI: matrix-assisted laser desorption ionization mass spectrometry; BDG: serological test with β-D-glucan; CT-SCAN: Computerized Tomography SCAN; CXR: Chest X-Ray; GM-EIA: Galactomannan Enzyme Immunoassay; NBL: Non-Bronchoscopic Lavage; BAL: Broncho-Alveolar Lavage; AFT: antifungal therapy; OPC: Oropharyngeal Candidiasis.

## Data Availability

The data supporting the findings of this study are available upon reasonable request from the corresponding author.

## References

[1] Morey-Olivé M., Espiau M., Mercadal-Hally M., Lera-Carbasllo E., García-Patos V. (2020). Manifestaciones cutáneas en contexto del brote actual de enfermedad por coronavirus 2019. An. Pediatr. (Engl. Ed.).

[2] Seagle E.E., Jackson B.R., Lockhart S.R., Georgacopoulos O., Nunnally N.S., Roland J., Barter D.M., Johnston H.L., Czaja C.A., Kayalioglu H. (2022). The Landscape of Candidemia During the Coronavirus Disease 2019 (COVID-19) Pandemic. Clin. Infect. Dis..

[3] Machado M., Estévez A., Sánchez-Carrillo C., Guinea J., Escribano P., Alonso R., Valerio M., Padilla B., Bouza E., Muñoz P. (2022). Incidence of Candidemia Is Higher in COVID-19 versus Non-COVID-19 Patients, but Not Driven by Intrahospital Transmission. J. Fungi.

[4] Shishido A.A., Mathew M., Baddley J.W. (2022). Overview of COVID-19-Associated Invasive Fungal Infection. Curr. Fungal Infect. Rep..

[5] Szabo B.G., Lakatos B., Bobek I., Szabo E., Szlavik J., Vályi-Nagy I. (2021). Invasive fungal infections among critically ill adult COVID-19 patients: First experiences from the national centre in Hungary. J. Mycol. Med..

[6] Koukaki E., Rovina N., Tzannis K., Sotiropoulou Z., Loverdos K., Koutsoukou A., Dimopoulos G. (2022). Fungal Infections in the ICU during the COVID-19 Era: Descriptive and Comparative Analysis of 178 Patients. J. Fungi.

[7] Kayaaslan B., Eser F., Kalem A.K., Kaya G., Kaplan B., Kacar D., Hasanoglu I., Coskun B., Guner R. (2021). Post-COVID syndrome: A single-center questionnaire study on 1007 participants recovered from COVID-19. J. Med. Virol..

[8] Moser D., Biere K., Han B., Hoerl M., Schelling G., Choukér A., Woehrle T. (2021). COVID-19 Impairs Immune Response to Candida albicans. Front. Immunol..

[9] Ahmed N., Mahmood M.S., Ullah M.d.A., Araf Y., Rahaman T.I., Moin A.T., Hosen M.J. (2022). COVID-19-Associated Candidiasis: Possible Patho-Mechanism, Predisposing Factors, and Prevention Strategies. Curr. Microbiol..

[10] Hoenigl M., Seidel D., Sprute R., Cunha C., Oliverio M., Goldman G.H., Ibrahim A.S., Carvalho A. (2022). COVID-19-associated fungal infections. Nat. Microbiol..

[11] Tricco A.C., Lillie E., Zarin W., O’Brien K.K., Colquhoun H., Levac D., Moher D., Peters M.D., Horsley T., Weeks L. (2018). PRISMA extension for scoping reviews (PRISMA-ScR): Checklist and explanation. Ann. Intern. Med..

[12] Page M.J., Moher D., Bossuyt P.M., Boutron I., Hoffmann T.C., Mulrow C.D., Shamseer L., Tetzlaff J.M., Akl E.A., Brennan S.E. (2021). PRISMA 2020 explanation and elaboration: Updated guidance and exemplars for reporting systematic reviews. BMJ.

[13] Burns P.B., Rohrich R.J., Chung K.C. (2011). The Levels of Evidence and Their Role in Evidence-Based Medicine. Plast. Reconstr. Surg..

[14] Segrelles-Calvo G., de S. Araújo G.R., Llopis-Pastor E., Carrillo J., Hernández-Hernández M., Rey L., Melean N., Escribano I., Antón E., Zamarro C. (2021). Candida spp. co-infection in COVID-19 patients with severe pneumonia: Prevalence study and associated risk factors. Respir. Med..

[15] Jeong S., Lee N., Park Y., Kim J., Jeon K., Park M.-J., Song W. (2022). Prevalence and Clinical Impact of Coinfection in Patients with Coronavirus Disease 2019 in Korea. Viruses.

[16] Porto A.P.M., Borges I.C., Buss L., Machado A., Bassetti B.R., Cocentino B., Bicalho C.S., Carrilho C., Rodrigues C., Neto E. (2023). Healthcare-associated infections on the intensive care unit in 21 Brazilian hospitals during the early months of the coronavirus disease 2019 (COVID-19) pandemic: An ecological study. Infect. Control Hosp. Epidemiol..

[17] Nucci M., Barreiros G., Guimarães L.F., Deriquehem V.A.S., Castiñeiras A.C., Nouér S.A. (2021). Increased incidence of candidemia in a tertiary care hospital with the COVID-19 pandemic. Mycoses.

[18] Pemán J., Ruiz-Gaitán A., García-Vidal C., Salavert M., Ramírez P., Puchades F., García-Hita M., Alastruey-Izquierdo A., Quindós G. (2020). Fungal co-infection in COVID-19 patients: Should we be concerned?. Rev. Iberoam. Micol..

[19] Abdoli A., Falahi S., Kenarkoohi A. (2021). COVID-19-associated opportunistic infections: A snapshot on the current reports. Clin. Exp. Med..

[20] Chiurlo M., Mastrangelo A., Ripa M., Scarpellini P. (2021). Invasive fungal infections in patients with COVID-19: A review on pathogenesis, epidemiology, clinical features, treatment, and outcomes. New Microbiol..

[21] Rajendra Santosh A.B., Muddana K., Bakki S.R. (2021). Fungal Infections of Oral Cavity: Diagnosis, Management, and Association with COVID-19. SN Compr. Clin. Med..

[22] Arastehfar A., Hilmioğlu-Polat S., Daneshnia F., Pan W., Hafez A., Fang W., Liao W., Şahbudak-Bal Z., Metin D., Junior J.M. (2021). Clonal Candidemia Outbreak by Candida parapsilosis Carrying Y132F in Turkey: Evolution of a Persisting Challenge. Front. Cell Infect. Microbiol..

[23] Frías-De-León M.G., Pinto-Almazán R., Hernández-Castro R., García-Salazar E., Meza-Meneses P., Rodríguez-Cerdeira C., Arenas R., Conde-Cuevas E., Acosta-Altamirano G., Martínez-Herrera E. (2021). Epidemiology of Systemic Mycoses in the COVID-19 Pandemic. J. Fungi.

[24] Katz J. (2021). Prevalence of candidiasis and oral candidiasis in COVID-19 patients: A cross-sectional pilot study from the patients’ registry in a large health center. Quintessence Int..

[25] Coskun A.S., Durmaz Ş.Ö. (2021). Fungal Infections in COVID-19 Intensive Care Patients. Pol. J. Microbiol..

[26] Erami M., Mirhendi H., Momen-Heravi M., Hezaveh S.J.H., Ahsaniarani A.H., Sabet S.S., Aboutalebian S. (2022). A case of COVID-19-associated rhino-orbito-cerebral mucormycosis caused by Apophysomyces variabilis with a review of the literature. Front. Cell Infect. Microbiol..

[27] Salehi M., Ahmadikia K., Badali H., Khodavaisy S. (2020). Opportunistic Fungal Infections in the Epidemic Area of COVID-19: A Clinical and Diagnostic Perspective from Iran. Mycopathologia.

[28] Vitale R.G., Afeltra J., Seyedmousavi S., Giudicessi S.L., Romero S.M. (2022). An overview of COVID-19 related to fungal infections: What do we know after the first year of pandemic?. Braz. J. Microbiol..

[29] White P.L., Price J.S., Cordey A., Backx M. (2021). Molecular Diagnosis of Yeast Infections. Curr. Fungal Infect. Rep..

[30] Salehi M., Ahmadikia K., Mahmoudi S., Kalantari S., Jamalimoghadamsiahkali S., Izadi A., Kord M., Manshadi S., Seifi A., Ghiasvand F. (2020). Oropharyngeal candidiasis in hospitalised COVID-19 patients from Iran: Species identification and antifungal susceptibility pattern. Mycoses.

[31] Senok A., Alfaresi M., Khansaheb H., Nassar R., Hachim M., Al Suwaidi H., Almansoori M., Alqaydi F., Afaneh Z., Mohamed A. (2021). Coinfections in Patients Hospitalized with COVID-19: A Descriptive Study from the United Arab Emirates. Infect. Drug Resist..

[32] Sang L., Xi Y., Lin Z., Pan Y., Song B., Li C., Zheng X., Zhong M., Jiang L., Pan C. (2021). Secondary infection in severe and critical COVID-19 patients in China: A multicenter retrospective study. Ann. Palliat. Med..

[33] Mastrangelo A., Germinario B.N., Ferrante M., Frangi C., Li Voti R., Muccini C., Ripa M., COVID-BioB Study Group (2021). Candidemia in Coronavirus Disease 2019 (COVID-19) Patients: Incidence and Characteristics in a Prospective Cohort Compared with Historical Non–COVID-19 Controls. Clin. Infect. Dis..

[34] Amorim dos Santos J., Normando A.G.C., Carvalho da Silva R.L., Acevedo A.C., De Luca Canto G., Sugaya N., Santos-Silva A.R., Guerra E.N.S. (2021). Oral Manifestations in Patients with COVID-19: A 6-Month Update. J. Dent. Res..

[35] Roudbary M., Kumar S., Kumar A., Černáková L., Nikoomanesh F., Rodrigues C.F. (2021). Overview on the Prevalence of Fungal Infections, Immune Response, and Microbiome Role in COVID-19 Patients. J. Fungi.

[36] Denny S., Abdolrasouli A., Elamin T., Gonzalo X., Charani E., Patel A., Donaldson H., Hughes S., Armstrong-James D., More L. (2021). A retrospective multicenter analysis of candidaemia among COVID-19 patients during the first UK pandemic wave. J. Infect..

[37] Norberg C.M., Norberg P.R., Norberg A., Lopes de Matos A.A., Sanches F., Manhães F., Mangiavacchi B., Faial L. (2021). Candida infections associated with COVID-19: An underestimated risk. WJPPS.

[38] Samaranayake L.P., Seneviratne C.J., Fakhruddin K.S. (2022). Coronavirus disease 2019 (COVID-19) vaccines: A concise review. Oral. Dis..

[39] Brandi N., Ciccarese F., Balacchi C., Rimondi M.R., Modolon C., Sportoletti C., Capozzi C., Renzulli M., Paccapelo A., Castelli A. (2022). Co-Infections and Superinfections in COVID-19 Critically Ill Patients Are Associated with CT Imaging Abnormalities and the Worst Outcomes. Diagnostics.

[40] Rafat Z., Ramandi A., Khaki P.A., Ansari S., Ghaderkhani S., Haidar H., Tajari F., Roostaei D., Ghazvini R., Hashemi S. (2022). Fungal and bacterial co-infections of the respiratory tract among patients with COVID-19 hospitalized in intensive care units. Gene Rep..

[41] Ayalon O., Cohen M.J., Orenbuch-Harroch E., Sviri S., van Heerden P.V., Korem M. (2022). Invasive fungal infections in critically ill COVID-19 patients in a large tertiary university hospital in Israel. J. Crit. Care.

[42] Soltani S., Zakeri A., Zandi M., Kesheh M.M., Tabibzadeh A., Dastranj M., Faramarzi S., Didehdar M., Hafezi H., Hosseini P. (2021). The Role of Bacterial and Fungal Human Respiratory Microbiota in COVID-19 Patients. Biomed. Res. Int..

[43] Kamali Sarvestani H., Mahmoudi S., Afarinesh Khaki P., Ansari S., Ghaderkhani S., Roostaei D., Ghazvini R., Hashemi S., Rafat Z., Abollahi A. (2022). Epidemiology, risk factors, species distribution, and antifungal susceptibility of candidemia among hospitalized patients with COVID-19. Curr. Med. Mycol..

[44] Kubin C.J., McConville T.H., Dietz D., Zucker J., May M., Nelson B., Istorico E., Bartram L., Small-Saunders J., Sobieszczyk M. (2021). Characterization of Bacterial and Fungal Infections in Hospitalized Patients with Coronavirus Disease 2019 and Factors Associated with Health Care-Associated Infections. Open Forum Infect. Dis..

[45] Cataldo M.A., Tetaj N., Selleri M., Marchioni L., Capone A., Caraffa E., Di Caro A., Petrosillo N., INMICOVID-19 Co-infection Group (2020). Incidence of bacterial and fungal bloodstream infections in COVID-19 patients in intensive care: An alarming “collateral effect”. J. Glob. Antimicrob. Resist..

[46] Agrifoglio A., Cachafeiro L., Figueira J.C., Añón J.M., García de Lorenzo A. (2020). Critically ill patients with COVID-19 and candidaemia: We must keep this in mind. J. Mycol. Med..

[47] Hughes S., Troise O., Donaldson H., Mughal N., Moore L.S.P. (2020). Bacterial and fungal coinfection among hospitalized patients with COVID-19: A retrospective cohort study in a UK secondary-care setting. Clin. Microbiol. Infect..

[48] Antinori S., Bonazzetti C., Gubertini G., Capetti A., Pagani C., Morena V., Rimoldi S., Galimberti L., Sarzi-Puttini P., Ridolfo A. (2020). Tocilizumab for cytokine storm syndrome in COVID-19 pneumonia: An increased risk for candidemia?. Autoimmun. Rev..

[49] Papadimitriou-Olivgeris M., Kolonitsiou F., Kefala S., Spiliopoulou A., Aretha D., Bartzavali C., Siapika A., Marangos M., Fligou F. (2022). Increased incidence of candidemia in critically ill patients during the Coronavirus Disease 2019 (COVID-19) pandemic. Braz. J. Infect. Dis..

[50] Baddley J.W., Thompson G.R., Chen S.C.-A., White P.L., Johnson M.D., Nguyen M.H., Schwartz I., Spec A., Ostrosky-Zeichner L., Jackson B. (2021). Coronavirus Disease 2019–Associated Invasive Fungal Infection. Open Forum Infect. Dis..

[51] Macauley P., Martin A., Epelbaum O. (2020). Corticosteroids in the Treatment of Severe COVID-19 Lung Disease: The Pulmonology Perspective from the First United States Epicenter. Int. J. Infect. Dis..

[52] Basile K., Halliday C., Kok J., Chen S.C.-A. (2022). Fungal Infections Other Than Invasive Aspergillosis in COVID-19 Patients. J. Fungi.

[53] Elbaz M., Korem M., Ayalon O., Wiener-Well Y., Shachor-Meyouhas Y., Cohen R., Bishara J., Atamna A., Brosh-Nissimov T., Maaravi N. (2022). Invasive Fungal Diseases in Hospitalized Patients with COVID-19 in Israel: A Multicenter Cohort Study. J. Fungi.

[54] Santosh A.B.R., Muddana K., Bakki S.R. (2021). Response to Commentary: Fungal Infections of Oral Cavity: Diagnosis, Management, and Association with COVID-19. SN Compr. Clin. Med..

[55] Arastehfar A., Carvalho A., Nguyen M.H., Hedayati M.T., Netea M.G., Perlin D.S., Hoenigl M. (2020). COVID-19-Associated Candidiasis (CAC): An Underestimated Complication in the Absence of Immunological Predispositions?. J. Fungi.

[56] Lamers M.M., Beumer J., Vaart J., Knoops K., Puschhof J., Breugem T.I., Ravelli R., Schayck J.P., Mykytyn A., Duimel H. (2020). SARS-CoV-2 productively infects human gut enterocytes. Science.

[57] Torres-Estrella C.U., Reyes-Montes Mdel R., Duarte-Escalante E., Sierra Martínez M., Frías-De-León M.G., Acosta-Altamirano G. (2022). Vaccines Against COVID-19: A Review. Vaccines.

[58] Acosta-Altamirano G. (2021). Nasal Mask: An Alternative to Prevent Contagion during Essential Activities. Biomed. J. Sci. Tech. Res..

[59] Feng Y., Ling Y., Bai T., Xie Y., Huang J., Li J., Xiong W., Yang D., Chen R., Lu F. (2020). COVID-19 with Different Severities: A Multicenter Study of Clinical Features. Am. J. Respir. Crit. Care Med..

[60] Wu C., Chen X., Cai Y., Xia J., Zhou X., Xu S., Huang H., Zhang L., Zhou X., Du C. (2020). Risk Factors Associated with Acute Respiratory Distress Syndrome and Death in Patients with Coronavirus Disease 2019 Pneumonia in Wuhan, China. JAMA Intern. Med..

[61] Amorim dos Santos J., Normando A.G.C., Carvalho da Silva R.L., Acevedo A.C., De Luca Canto G., Sugaya N., Santos-Silva A.R., Guerra E.N.S. (2021). Oral Manifestations in Patients with COVID-19: A Living Systematic Review. J. Dent. Res..

[62] Denny S., Rawson T.M., Hart P., Satta G., Abdulaal A., Hughes S., Gilchrist M., Mughal N., Moore L. (2021). Bacteraemia variation during the COVID-19 pandemic; A multi-centre UK secondary care ecological analysis. BMC Infect. Dis..

[63] Arastehfar A., Daneshnia F., Najafzadeh M.J., Hagen F., Mahmoudi S., Salehi M., Zarrinfar H., Namvar Z., Zareshahrabadi Z., Khodavaisy S. (2020). Evaluation of Molecular Epidemiology, Clinical Characteristics, Antifungal Susceptibility Profiles, and Molecular Mechanisms of Antifungal Resistance of Iranian Candida parapsilosis Species Complex Blood Isolates. Front. Cell Infect. Microbiol..

[64] Arastehfar A., Daneshnia F., Farahyar S., Fang W., Salimi M., Salehi M., Hagen F., Weihua P., Roudbary M., Boekhout T. (2019). Incidence and spectrum of yeast species isolated from the oral cavity of Iranian patients suffering from hematological malignancies. J. Oral. Microbiol..

[65] Omrani A.S., Koleri J., Ben Abid F., Daghfel J., Odaippurath T., Peediyakkal M.Z., Baiou A., Sarsak E., Elayana M., Kaleeckal A. (2021). Clinical characteristics and risk factors for COVID-19-associated Candidemia. Med. Mycol..

[66] Erami M., Raiesi O., Momen-Heravi M., Getso M.I., Fakhrehi M., Mehri N., Yarahmadi M., Amiri S., Raissi V., Hashemi S. (2022). Clinical impact of Candida respiratory tract colonization and acute lung infections in critically ill patients with COVID-19 pneumonia. Microb. Pathog..

[67] Bishburg E., Okoh A., Nagarakanti S.R., Lindner M., Migliore C., Patel P. (2021). Fungemia in COVID-19 ICU patients, a single medical center experience. J. Med. Virol..

[68] Cannon R.D. (2022). Oral Fungal Infections: Past, Present, and Future. Front. Oral Health..

[69] Suleyman G., Alangaden G.J. (2021). Nosocomial Fungal Infections: Epidemiology, Infection Control, and Prevention. Infect. Dis. Clin. N. Am..

[70] Adzic-Vukicevic T., Velickovic J., Radovanovic-Spurnic A., Velickovic D., Milenkovic S., Petrovic F., Micic J., Dragutinovic N. (2022). Fatal invasive candidiasis in COVID-19 patient with severe bleeding and extensively drug-resistant *Klebsiella enterobacter*. J. Infect. Dev. Ctries..

[71] Ezeokoli O.T., Pohl C.H. (2020). Opportunistic pathogenic fungal co-infections are prevalent in critically ill COVID-19 patients: Are they risk factors for disease severity?. S. Afr. Med. J..

